# A Retrospective Study Comparing the Surgical Results of Platysma Myocutaneous Flap, Buccal Pad of Fat, and Nasolabial Flap for Reconstruction After Fibrotomy in Cases of Oral Submucous Fibrosis

**DOI:** 10.7759/cureus.60297

**Published:** 2024-05-14

**Authors:** Wahengbam Tulsidas Singh, Nitin Bhola, Deepak N Singh, Anchal Agarwal, Potsangbam Aparna Devi, Kiran Kumar Aheibam

**Affiliations:** 1 Department of Oral and Maxillofacial Surgery, Dental College, Regional Institute of Medical Sciences, Imphal, IND; 2 Department of Oral and Maxillofacial Surgery, Sharad Pawar Dental College and Hospital, Datta Meghe Institute of Higher Education and Research, Sawangi (Meghe), Wardha, IND; 3 Department of Oral Medicine and Radiology, Dental College, Regional Institute of Medical Sciences, Imphal, IND; 4 Department of Oral and Maxillofacial Surgery, Sharad Pawar Dental College, Datta Meghe Institute of Higher Education and Research, Sawangi (Meghe), Wardha, IND; 5 Department of Oral Pathology and Microbiology, Dental College, Regional Institute of Medical Sciences, Imphal, IND; 6 Department of Conservative and Endodontics, Dental College, Regional Institute of Medical Sciences, Imphal, IND

**Keywords:** platysma myocutaneous flap, oral submucous fibrosis, nasolabial flap, fibrotomy, buccal pad of fat flap

## Abstract

Purpose: To evaluate the effectiveness of nasolabial flap (NLF), a buccal pad of fat flap (BFP), and platysma myocutaneous flap (PMF) for reconstruction following fibrotomy for individuals with oral submucous fibrosis (OSMF).

Material and method: A retrospective study was conducted among patients diagnosed with grade III and IV OSMF in the Department of Oral and Maxillofacial Surgery at Sharad Pawar Dental College between January 2016 and August 2018. The essential patient information was obtained from the Medical Record Department (MRD) at Acharya Vinoba Bhave Rural Hospital (AVBRH), Datta Meghe Institute of Medical Sciences (DMIMS) Sawangi (Meghe) Wardha. The patients were categorized into three groups: the NLF, the BFP, and the PMF groups. Each group had 16 patients, and factors such as interincisal width, diminished burning sensation in the mouth, inter-commissure distance, and flap necrosis were compared pre- and post-operatively. Student's unpaired t-test and chi-square test were employed for statistical analysis.

Result: Mean interincisal mouth-opening increased from pre-operative 4.79 to 41.42 mm post-operatively in the NLF group, BFP group from 6 to 39.42 mm and in the PMF group from 9.26 to 39.34 mm with p value=0.0001. NLF group showed complete and partial resolution of the burning sensation of the mouth at 93.75% and 6.25%, BFP at 62.25% and 32.75% while in PMF it was 68.5% and 31.25% respectively. One year postoperatively 3.28 mm increase in inter-commissure width was observed in the NLF group with a marginal increase in the PMF group and a negligible increase in the BFP group. 18.75% partial flap necrosis was seen in BFP, 18.75% in the PMF group, and 6.25% in the NFL group.

Conclusion: All the flaps are efficacious in treating OSMF, however, NLF stands ahead with its higher reliability owing to its excellent blood supply.

## Introduction

Oral submucous fibrosis (OSMF), a chronic oral mucosal disease, predominantly impacts populations of Indian descent and Indian-origin individuals who reside abroad rather than in India. Additionally, it has an impact on other Asians and, less commonly, Europeans [1].

The initial characterization of OSMF was documented by Schwartz in 1952. The prevalence of this precancerous condition is widely acknowledged across the Indian subcontinent [2]. OSMF is a persistent and gradual ailment that impacts several regions of the oral cavity, occasionally extending to the pharynx. The condition is consistently associated with an inflammatory reaction in the juxta-epithelial layer, accompanied by a fibro-elastic change in the lamina propria. Epithelial degeneration leads to rigidity of the oral mucosa, leading to trismus and difficulty eating. However, it is occasionally accompanied by the formation of vesicles [3,4].

The etiology of OSMF is related to the areca nut, which is also known as betel nut or supari. Arecoline, an alkaloid present in areca nut, stimulates the synthesis of collagen and the growth of fibroblasts. The areca nut also contains flavonoids, catechins, and tannins, which stabilize the collagen fibrils and prevent collagenase from breaking them down [5]. There are non-surgical and surgical modes of management. Non-surgical treatment includes intralesional injection of the hyaluronidase, hydrocortisone, placental extract, or triamcinolone acetonamide. This in turn is supplemented with the addition of multivitamins and iron [6]. Non-surgical treatment is limited to only early cases of OSMF.

The choice of treatment for advanced OSMF cases, where the mouth opening is minimal, is solely surgical. Post fibrotomy many types of flaps can be interposed to prevent re-fibrosis and treatment failure. In our study, we have compared the efficacy of the nasolabial flap (NLF), buccal pad of fat flap (BFP), and platysma myocutaneous flap (PMF) for reconstruction following fibrotomy in OSMF cases. We have hypothesized that the nasolabial flap could be a better choice of reconstruction following fibrotomy in terms of reliability and efficacy.

## Materials and methods

All the cases of grade III and IV OSMF categorized based on the classification given by Khanna and Andrade in 1995 [7] and reported to the Department of Oral and Maxillofacial Surgery, Sharad Pawar Dental College between January 2016 to August 2018 were selected for the study. Necessary data/records of the patients were collected from the MRD, Acharya Vinoba Bhave Rural Hospital Sawangi (Meghe) Wardha.

The study was carried out following the acquisition of approval from the Institutional Ethical Committee of Datta Meghe Institute of Medical Sciences Sawangi (Meghe) Wardha (DMIMS(DU)/IEC/2018-19/7714). A total of 48 patients, 16 each in NFL, BFP, and PMF were included. Those patients operated combining with any other flaps were eliminated. Only those fulfilling the above criteria and willing to follow-up were included.

The patients were evaluated in the following variables in all the groups pre-operatively and one year postoperatively in terms of mouth opening, reduction in burning sensation of the mouth, inter-commissure distance, and flap necrosis. Routine investigations were required before surgery was done. Under standard aseptic conditions, either blind intubation or a fiberoptic bronchoscope was utilized for intubation of the patients for general anesthesia administration.

To prevent injury, Stenson's duct was identified before making the incision. Using an electrosurgical knife, incisions were created at the linea alba level, extending from the corner of the mouth to the soft palate or retromandibular raphae. A Y-shaped incision was made anteriorly to stop the mucosal rip at the mouth's corner. The bands were fibrotomized by blunt dissection, which was done with either the finger or mosquito forceps. The interincisal opening was then noted after that. The incision was utilized to access the coronoid processes, and a bilateral coronoidectomy or coronoidotomy was performed using a malleable retractor to preserve the underlying structures. The third molars in the mandible and maxilla were removed.

Bi-winged NLF was elevated bilaterally in the superficial muscular aponeurotic system (SMAS) in the NLF group to perform NLF grafting from the leading edge of the nasolabial fold to one centimeter below the mouth's angle. The pedicle's diameter was around 2 to 2.5 cm, and it was located 1 cm lateral to the mouth's corner. The small, tension-free transbuccal tunnel was utilized to place the flap intraorally, near the mouth commissure. The flap's inferior wing was separated and sutured to the defect's anterior borders, while the superior wing was secured to the defect's posterior edges.

To avoid the minimal post-operative increase in inter-commissure width, the lateral aspect of the donor region was liberally undermined intraoperatively in the subcutaneous region. The buccal pad of fat in the BFP group was taken from the raw area's postero-superior edge, which was formed following the fibrotomy process, and extended up to the retromolar trigone. The average dimensions of the defect were measured to be roughly 3 to 5 cm in length and 4 to 6 cm in width, depending on its magnitude. The BFP was gradually removed until there was enough to cover the exposed area tension-free. Using interrupted sutures and 3-0 Vicryl, the flap was sutured to the defect. To completely rule out the chance of subsequent epithelialization, the entire defect was covered with BFP.

**Figure 1:**
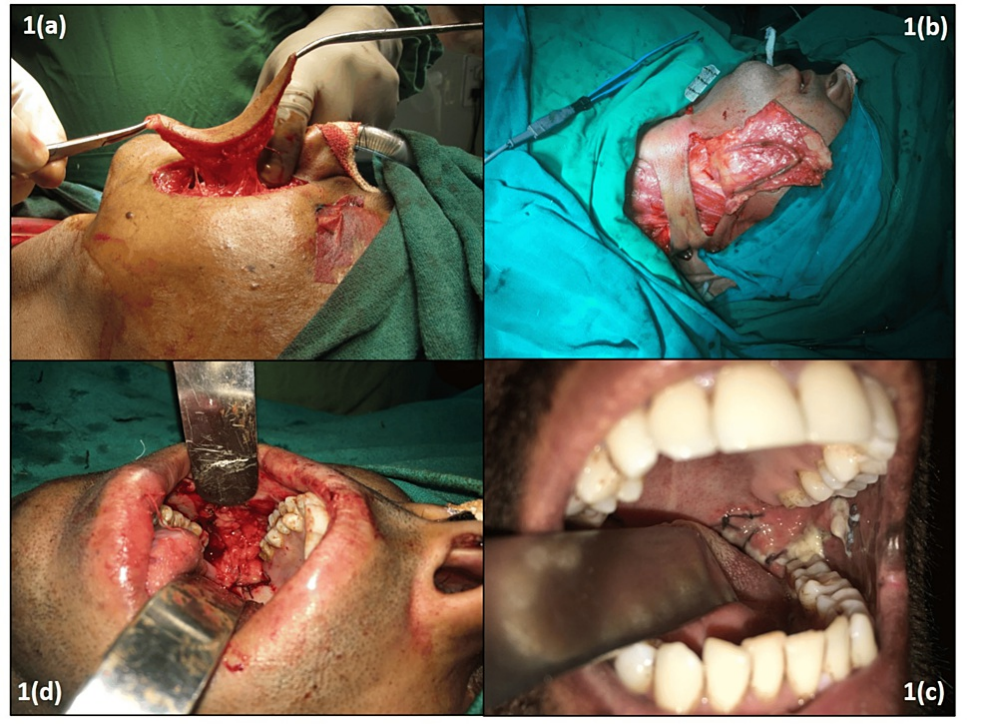
Intra-operative and post-operative images of different flap designs 1(a): Intra-operative image of nasolabial flap; 1(b): Intra-operative image of Platysma Myocutaneous Flap; 1(c): Post-operatuve image of Buccal Pad of Fat graft; 1(d): Intra-operative image of Buccal Pad of Fat graft

According to Baur's description, a superiorly based PMF was raised in the PMF group [8]. On the ipsilateral side, the skin paddle was delineated and the neck was hyperextended, extending down to the inferior border of the jaw. Figure 1 presents the intra-operative and post-operative images of three different flaps. 

Carefully inserted and dissected superficially to the platysma muscle, the superior incision was made and extended cephalic to the inferior border of the jaw. The platysma was also exposed inferiorly, and an incision was made in the skin near the inferior line of the skin paddle. The vascular supply for the superiorly based flap is provided by the submental branch of the facial artery, which originates from the submandibular gland approximately 1 to 2 cm beneath the lower margin of the jaw. Hence, it is advisable to refrain from conducting the dissection beyond that particular stage. A transverse incision was made in the platysma muscle, at least 2 centimeters below the edge of the skin paddle. The dissection was next performed on the subplatysmal plane, which extended in a cephalad direction to a position just below the inferior border of the jaw. The platysma can be transected vertically, anteriorly, and posteriorly to completely release the flap when both planes of dissection have reached full development. A soft tissue tunnel between one and two centimeters in size can be created to introduce the flap into the oral cavity once it has been mobilized. Adequate breadth of the tunnel is necessary to prevent strangulation and flap twisting, which may jeopardize the blood flow. To achieve a satisfactory esthetic outcome, the recipient and donor regions can be closed with minimum effort.

To prevent fluids from collecting at the donor site, a corrugated rubber drain was installed and removed after 48 hours. After a week, the sutures from the donor location were taken out. Following surgery, all patients were placed on Ryle's tube feeding for ten days and were given prophylactic antibiotics. After four days, mouth physiotherapy was initiated with the use of a wooden spatula and Hiester's jaw exerciser to prevent contracture and relapse.

Statistical analysis

Statistical Package for Social Sciences (SPSS) (IBM SPSS Statistics for Windows, Version 26.0, Armonk, NY, USA) was used for all statistical analysis. For statistical analysis, we employed the chi-square test and the Student's unpaired t-test to contrast the average values between the groups.

## Results

The most common age group affected with OMFS was between 26 to 35 years with male predominance (Tables 1, 2).

**Table 1:** Patients' age distribution NS – Non-significant difference at p≤0.05; BFP: Buccal pad of fat flap; NLF: Nasolabial flap; PMF: Platysma myocutaneous flap; The data has been represented as N, %

Age Group (yrs)	BFP	NLF	PMF
16-25 yrs	6(37.5%)	4(25%)	2(12.5%)
26-35 yrs	6(37.5%)	6(37.5%)	7(43.75%)
36-45 yrs	3(18.75%)	5(31.25%)	5(31.25%)
46-55 yrs	1(6.25%)	1(6.25%)	1(6.25%)
56-65 yrs	0(0%)	0(0%)	1(6.25%)
Total	16(100%)	16(100%)	16(100%)
Mean±SD	28.62±8.52	32.31±8.73	36.43±9.73
χ2-value	4.72, p=0.78, NS, p>0.05		

**Table 2:** Patient distribution by gender NS – Non-significant difference at p≤0.05; BFP: Buccal pad of fat flap; NLF: Nasolabial flap; PMF: Platysma myocutaneous flap; The data has been represented as N, %

Gender	BFP	NLF	PMF
Male	16(100%)	12(75%)	13(81.25%)
Female	0(0%)	4(25%)	3(18.75%)
Total	16(100%)	16(100%)	16(100%)
χ2-value	4.34, p=0.11, NS, p>0.05		

The average preoperative and one-year postoperative mouth opening in the NLF group was 4.79 and 41.42 mm respectively. The corresponding values of the BFP group were 6 and 39.42 mm and in the PMF group, it was 9.26 and 39.34 mm respectively with p=0.0001 (Table 3).

**Table 3:** Comparison of interincisal width (mm) in three groups pre- and post-operatively Student’s unpaired t-test; * indicates significant difference at p≤0.05; BFP: Buccal pad of fat flap; NLF: Nasolabial flap; PMF: Platysma myocutaneous flap; The data has been represented as Mean±SD

Group	Pre OT	1-year Post OT	Mean Difference	t-value	p-value
BFP	6.01±3.30	39.42±1.99	33.40±2.74	48.66	0.0001*
NLF	4.79±2.94	41.42±2.90	36.63±4.55	32.18	0.0001*
PMF	9.26±4.54	39.34±4.14	30.07±6.32	19.02	0.0001*

Table 4 depicts that in the NLF group, one-year postoperatively, complete resolution of the burning sensation of mouth was seen in 15 subjects (93.5%), and partial resolution was seen in one subject (6.25%). In the BFP group, complete resolution of the burning sensation of mouth was seen in 10 subjects (62.5%) and partial resolution was seen in six subjects (32.5%). In the PMF group, complete resolution of the burning sensation of mouth was seen in 11 subjects (68.75%) and partial resolution was seen in five subjects (31.25%), with overall p=0.32 (statistically non-significant). This finding signifies that the effect of reduction in the burning sensation of the mouth was higher when NLF was used to interpose after fibrotomy.

**Table 4:** Comparison of reduction in burning sensation of mouth in three groups pre- and six months post-operatively chi-square test; NS – Non-significant difference at p≤0.05; BFP: Buccal pad of fat flap; NLF: Nasolabial flap; PMF: Platysma myocutaneous flap; The data has been represented as N, %

	Pre-Op burning sensation of mouth	Post Op 1 year burning sensation of mouth			p-value
		Partial Resolution	Complete Resolution	No Response	
BFP	16(100%)	6(37.5%)	10(62.5%)	0(0%)	1.66 p=0.32, NS
NLF	16(100%)	1(6.25%)	15(93.75%)	0(0%)	
PMF	16(100%)	5(31.25%)	11(68.75%)	0(0%)	

The inter-commissure distance was increased by 3.28 mm in the NLF group from preoperative to one-year postoperative values being 41.03 and 44.31mm respectively, PMF group preoperative and postoperative inter-commissure were 41.87 and 42.25 mm respectively while BFP group showed not much changes in preoperative and one-year postoperative values (Table 5) with p< 0.05 (statistically significant in all the groups).

**Table 5:** Comparison of inter commissure distance (mm) in three groups pre- and post-operatively Student’s unpaired t-test; * indicates significant difference at p≤0.05; BFP: Buccal pad of fat flap; NLF: Nasolabial flap; PMF: Platysma myocutaneous flap; The data has been represented as Mean±SD

Group	Pre OT	1-year Post OT	Mean Difference	t-value	p-value
BFP	41.03±1.57	41.31±1.67	0.28±0.40	2.76	0.014*
NFL	41.56±1.89	44.78±2.10	3.21±1.23	10.40	0.0001*
PMF	41.87±1.86	42.25±1.87	0.37±0.53	2.81	0.013*

Partial flap necrosis among the BFP and PMF groups was 18.75% (N = 3 cases each) and in the NLF group, it was 6.25% (N=1) (Table 6).

**Table 6:** Comparison of flap necrosis in three groups BFP: Buccal pad of fat flap; NLF: Nasolabial flap; PMF: Platysma myocutaneous flap; The data has been represented as N, %

Flap Necrosis	BFP	NLF	PMF
Partial Flap Necrosis	3(18.75%)	1(6.25%)	3(18.75%)
Complete Flap Necrosis	0(0%)	0(0%)	0(0%)

## Discussion

The generalized condition of the oral mucosa is altered in OSMF, and the previous attempts at surgical intervention were unsuccessful because, after fibrotomy, the wound heals by secondary intention, leading to excessive rebound fibrosis that leads to severe fibrosis and contracture [5]. Yen clarified the necessity of the first closure of a fibrotomy defect and identified the rebound fibrosis process in surgical OSMF surgical procedures [9]. Since then, bilateral fibrotomy has been the surgical therapy for OSMF. The fibrotomy defect is subsequently closed using the appropriate flap, graft tissue, or alloplastic material. Many flaps, including temporal myofascial flaps, PMF, palatal island flaps, bilateral tongue flaps, full thickness or split thickness free skin grafts, BFP, bilateral radial forearms, and anterolateral thigh free flaps, have been used with varying degrees of success for the closure of the defect [5]. Caniff et al. proposed the utilization of temporal myotomy or coronoidectomy as a potential treatment for alleviating persistent trismus resulting from the subsequent atrophic changes in the temporalis muscle tendon caused by the condition. After performing a bilateral coronoidectomy, there is a significant improvement in mouth opening during the surgical procedure if it remains below 35 mm after the fibrosis is released [10].

The common age group frequently affected with OSMF was found among 26- to 35-year-old subjects with male predominance. These findings were almost similar to the study conducted by Angadi and Rekha (2010), which found that the age group 20-30 years was most widely affected by OSMF with male preponderance [2]. When the nasolabial flap was transposed the interincisal width increased significantly from a mean of 14 (range: 3 to 23) mm to 41 (range: 23 to 55) mm at the end of six months and persisted without relapse in the study conducted by Borle et al. [11]. Akin to their study in our study when the NLF was interposed the interincisal width increased from mean 4.79±2.9 to post-operatively 41.42±2.9 with a follow-up of minimum one-year and has shown a high significant p-value (p=0.0001).

In the present study there was a substantial difference in the mean interincisal width in the BFP group pre-operatively (6.01±3.3) and postoperatively (39.4±1.99), P=0.0001. Similarly, in the study conducted by Rai et al., the mean pre- and post-operative mouth opening in the BFP group were 12 mm and 29 mm respectively [12]. Bande et al. found that the mean mouth opening pre-operatively and post-operatively after one-year follow-up among individuals reconstructed with NLF were 12 (range: 3 to 14) mm and 40 mm and those who were treated with PMF were 11 (range: 3 to 13) and 41mm. The difference in mouth opening pre- and post-operatively was significant in both groups (p<0.01) [13]. In our study, the NLF group had mean interincisal width pre-operatively (4.79±2.9 mm) and post-operatively (41.42±2.9 mm), and in the PMF group pre-operatively (9.26±4.54) and post-operatively (39.34±4.14).

The difference in mouth opening pre- and post-operatively was significant in both the groups goes in hand with their findings (p=0.0001). Further, Borle et al. documented that the pre-operative inter-commissure width among those treated with NLF was 46 mm and increased to 49 mm in two years follow-up [11]. Almost similar findings were found in our study with the pre-operative intercommissure distance in patients treated with NLF being 41.56 mm and postoperatively after one-year being 44.78mm. In yet another investigation conducted by Rai et al., the pre-operative inter-commissure width among those in the NLF group was 42 mm and increased to 45 mm in one-year follow-up [12]. They also found that there was no significant change in inter-commissure width in the patients treated with a buccal pad of fat. These findings go in hand with our study where we found that there is an observable change in the inter-commissure width in patients treated with NLF in one-year follow-up and in those treated with BFP did not show much significant change.

The nasolabial flap has a random pattern depending on the subcutaneous vascular supply from the transverse face and angular veins. It's a straightforward, reliable flap. If donor site care is taken, donor site morbidity is minimal. In cases of OSMF, it successfully addresses both the anterior and posterior defects of fibrotomy. According to Ioannides and Fossion (1991), a small proportion of patients may encounter extraoral scarring, infections, flap necrosis of varying degrees, and wound dehiscence [14]. The absence of proper suturing might lead to the presence of asymmetries at the nasolabial fold area [12].

BFP obtained via intraoral surgery is straightforward, painless, and has no adverse effects on the donor site. BFP has a capacity of 9.6 ml on average (range: 8.3 to 11.9 ml). This can be used to cover defects up to three to five centimeters in size without endangering the vascular supply [15]. However, complications might arise from BFP. In chronic cases, it can cause significant atrophy, and when the anterior reach is insufficient, it can leave a raw area that heals secondary to the original cause, which can lead to recurrence [16,17].

In our study, the partial flap necrosis for the BFP group was 18.75% (N=3) and the main reason was forceful anterior coverage by pulling the flap to reach the anterior defect. Another reason could be due to less availability of the BFP owing to poor nourishment. Borle et al. reported in their study that NLF showed partial flap necrosis of 8.5% (N=4) [11]. Close to their findings, NLF had 6.25% (N=1) flap necrosis in our findings. Further, they found that while using NLF, 41 out of 47 subjects reported a decrease in the burning sensation. In the current study, unlike their findings, when NLF was used 15 out of 16 patients had complete resolution of the burning sensation and one of them had partial resolution of the burning sensation of the mouth.

According to Ariyan (1997), PMF was used to maintain a skin transplant following skin sloughing in facial reconstruction [18]. The literature has documented skin paddle sizes ranging from 5-10 cm to 7-14 cm [19]. To improve the chances of survival, Coleman et al. (1983) suggested creating a big skin paddle with several perforators that are at least 5 cm wide [20]. Although small skin paddles up to 5 cm were successfully utilized in this instance, small skin paddles lack enough perforators for adequate skin perfusion. The blue dark flap is an indication of venous congestion. This is a common observation, particularly in the flap version that is superiorly based and has inadequate venous drainage through the submental vein [20,21].

Venous congestion was typically self-limiting as reported by Bande et al., and the flap's long-term survival was achieved. Employing a suction drain at the donor region prevented the formation of hematomas. Partial necrosis was found in 8.51% of the 47 OSMF cases treated with PMF [22]. In contrast to their study, ours found that 18.75% of partial flap necrosis and venous congestion were typically self-limiting, resulting in the flap's long-term survival. When cancer is diagnosed at an early stage, treatment is often more effective, and the patient has a greater chance of a full recovery [23].

Clinical significance

The clinical significance of nasolabial flap reconstruction in OSMF lies in its potential to improve mouth opening, restore function, and enhance the quality of life for affected individuals. However, like any surgical intervention, weighing the benefits against potential risks is crucial as considering individual patient factors when determining the most appropriate treatment approach. The buccal pad of fat plays a significant role in OSMF treatment outcomes by providing tissue augmentation, vascularized graft material, scar reduction, and minimal donor site morbidity. Its utilization can improve functional and aesthetic outcomes, ultimately enhancing the quality of life for individuals affected by OSMF. The clinical significance of the platysma myocutaneous flap in OSMF lies in its ability to provide durable coverage of defects, deliver vascularized tissue for enhanced healing, improve functional outcomes, achieve favorable cosmetic results, and support rehabilitation efforts. When appropriately selected and performed, this reconstructive technique can significantly improve the overall outcomes and quality of life for individuals affected by OSMF.

Strengths and limitations

Retrospective studies are typically less resource-intensive compared to prospective studies since they utilize existing data. This makes them a cost-effective way to investigate the effectiveness of different surgical approaches. However, the major limitation of retrospective studies is that they often suffer from selection bias as they rely on existing data. Patients may have been selected for each surgical method based on various factors such as surgeon preference, patient preference, or severity of OSMF, which could introduce bias into the results. Also, the findings of this study may not be generalizable to all patients with OSMF, as they were based on a specific patient population treated at a particular institution or by a specific group of surgeons.

## Conclusions

This study was conducted in the quest to find the most suitable flap among NFL, BFP, and PMF groups or reconstruction following fibrotomy in OSMF patients. The surgical outcome of all the flaps was quite encouraging and all the flaps have shown their efficacy to treat OSMF. Nevertheless, the NFL surpasses the BFP and PMF due to its superior dependability and consistent outcomes achieved through repeated use. This is linked to its reliable vascularity, which is derived from multiple nearby vessels.

## References

[1] Pillai R, Balaram P, Reddiar KS (1992). Pathogenesis of oral submucous fibrosis. Relationship to risk factors associated with oral cancer. Cancer.

[2] Angadi PV, Rekha KP (2011). Oral submucous fibrosis: a clinicopathologic review of 205 cases in Indians. Oral Maxillofac Surg.

[3] Pindborg JJ, Sirsat SM (1966). Oral submucous fibrosis. Oral Surg Oral Med Oral Pathol.

[4] Sirsat SM, Khanolkar VR (1962). Submucous fibrosis of the palate and pillars of the fauces. Indian J Med Sci.

[5] Lambade P, Meshram V, Thorat P, Dawane P, Thorat A, Rajkhokar D (2016). Efficacy of nasolabial flap in reconstruction of fibrotomy defect in surgical management of oral submucous fibrosis: a prospective study. Oral Maxillofac Surg.

[6] Saravanan K, Narayanan V (2012). The use of buccal fat pad in the treatment of oral submucous fibrosis: a newer method. Int J Dent.

[7] Khanna JN, Andrade NN (1995). Oral submucous fibrosis: a new concept in surgical management. Report of 100 cases. Int J Oral Maxillofac Surg.

[8] Baur DA, Williams J, Alakaily X (2014). The platysma myocutaneous flap. Oral Maxillofac Surg Clin North Am.

[9] Yen DJ (1982). Surgical treatment of submucous fibrosis. J Oral Surg.

[10] Canniff JP, Harvey W, Harris M (1986). Oral submucous fibrosis: its pathogenesis and management. Br Dent J.

[11] Borle RM, Nimonkar PV, Rajan R (2009). Extended nasolabial flaps in the management of oral submucous fibrosis. Br J Oral Maxillofac Surg.

[12] Rai A, Datarkar A, Rai M (2014). Is buccal fat pad a better option than nasolabial flap for reconstruction of intraoral defects after surgical release of fibrous bands in patients with oral submucous fibrosis? A pilot study: a protocol for the management of oral submucous fibrosis. J Craniomaxillofac Surg.

[13] Bande CR, Datarkar A, Khare N (2013). Extended nasolabial flap compared with the platysma myocutaneous muscle flap for reconstruction of intraoral defects after release of oral submucous fibrosis: a comparative study. Br J Oral Maxillofac Surg.

[14] Ioannides C, Fossion E (1991). Nasolabial flap for the reconstruction of defects of the floor of the mouth. Int J Oral Maxillofac Surg.

[15] Stuzin JM, Wagstrom L, Kawamoto HK, Baker TJ, Wolfe SA (1990). The anatomy and clinical applications of the buccal fat pad. Plast Reconstr Surg.

[16] Gupta D, Sharma SC (1988). Oral submucous fibrosis--a new treatment regimen. J Oral Maxillofac Surg.

[17] Tideman H, Bosanquet A, Scott J (1986). Use of the buccal fat pad as a pedicled graft. J Oral Maxillofac Surg.

[18] Ariyan S (1997). The transverse platysma myocutaneous flap for head and neck reconstruction. Plast Reconstr Surg.

[19] Kamath VV (2015). Surgical interventions in oral submucous fibrosis: a systematic analysis of the literature. J Maxillofac Oral Surg.

[20] Coleman JJ 3rd, Jurkiewicz MJ, Nahai F, Mathes SJ (1983). The platysma musculocutaneous flap: experience with 24 cases. Plast Reconstr Surg.

[21] Uehara M, Helman JI, Lillie JH, Brooks SL (2001). Blood supply to the platysma muscle flap: an anatomic study with clinical correlation. J Oral Maxillofac Surg.

[22] Bande C, Joshi A, Gawande M, Tiwari M, Rode V (2018). Utility of superiorly based platysma myocutaneous flap for reconstruction of intraoral surgical defects: our experience. Oral Maxillofac Surg.

[23] Dixit A, Parekh NH, Anand R, Kamal N, Badiyani BK, Kumar A, Obulareddy VT (2023). A study to assess the awareness of adults about precancerous and cancerous lesions and the associated risk factors. J Pharm Bioallied Sci.

